# Increased 4-Hydroxynonenal Formation Contributes to Obesity-Related Lipolytic Activation in Adipocytes

**DOI:** 10.1371/journal.pone.0070663

**Published:** 2013-08-05

**Authors:** Ximei Zhang, Zhigang Wang, Jiaxin Li, Dongfang Gu, Songtao Li, Chen Shen, Zhenyuan Song

**Affiliations:** 1 Department of Kinesiology and Nutrition, University of Illinois at Chicago, Chicago, Illinois, United States of America; 2 Department of Histology and Embryology, Harbin Medical University, Harbin, Heilongjiang, China; 3 Department of Pathology, University of Illinois Medical Center, Chicago, Illinois, United States of America; Virginia Commonwealth University, United States of America

## Abstract

Oxidative stress in adipose tissue plays an etiological role in a variety of obesity-related metabolic disorders. We previously reported that increased adipose tissue 4-hydroxynonenal (4-HNE) contents contributed to obesity-related plasma adiponectin decline in mice. In the present study, we investigated the effects of intracellular 4-HNE accumulation on lipolytic response in adipocytes/adipose tissues and underlying mechanisms. In both fully-differentiated 3T3-L1 and primary adipocytes, a 5-hour 4-HNE exposure elevated lipolytic reaction in a dose-dependent manner at both basal and isoproterenol-stimulated conditions, evidenced by significantly increased glycerol and fatty acids releases. This conclusion was corroborated by the comparable observations when the minced human visceral adipose tissues were used. Mechanistic investigations revealed that 4-HNE-stimulated lipolytic activation is multifactorial. 4-HNE exposure quickly increased intracellular cyclic AMP (cAMP) level, which was concomitant with increased phosphorylations of protein kinase A (PKA) and its direct downstream target, hormone sensitive lipase (HSL). Pre-incubation with H89, a potent PKA inhibitor, prevented 4-HNE stimulated glycerol release, suggesting that enhanced lipolytic action in response to 4-HNE increase is mediated mainly by cAMP/PKA signal pathway in adipocytes. In addition to activating cAMP/PKA/HSL pathway, 4-HNE exposure also suppresses AMP-activated protein kinase (AMPK), a suppressive pathway for lipolysis, measured by both Western blotting for phosphorylated form of AMPK and ELISA for enzyme activity. Furthermore, 5-Aminoimidazole-4-carboxamide 1-beta-D-ribofuranoside (AICAR), a pharmacological AMPK activator, alleviated 4-HNE-induced lipolysis, suggesting that AMPK suppression also contributes to 4-HNE elicited lipolytic response. In conclusion, our findings indicate that increased intracellular 4-HNE accumulation in adipocytes/adipose tissues contributes to obesity-related lipolytic activation.

## Introduction

Adipose tissue plays a critical role in the regulation of whole body energy homeostasis. Excess energy is stored in adipocytes in the form of triglycerides (TG) and mobilized via a process named lipolysis in the form of free fatty acids (FFAs) and glycerol, which are used for energy requirement of other organs. The pathways controlling these pathways are highly regulated. Dysregulated lipolytic reactions may result in elevated levels of circulating FFAs, one of the major contributors for the development of insulin resistance in obesity and diabetes mellitus.

A variety of hormones regulate lipolytic process in adipose tissue and general intracellular regulatory systems are well characterized [1], [2]. The major hormones stimulating lipolysis are catecholamines. Catecholamines stimulation increases intracellular cAMP production and subsequent activation of cAMP-dependent PKA, followed by the phosphorylation of HSL and its translocation to the lipid droplet surface, a step for the lipase to access its triacylglycerol substrates [1]. Moreover, catecholamines-induced the MEK- extracellular signal-regulated kinases 1 and 2 (ERK1/2) pathway activation represents another important signaling pathway that modulates lipolysis [2]. In addition to these conventional pathways, recent studies also demonstrated that activation of AMPK can suppress HSL phosphorylation/activation, thereby exerting anti-lipolytic effects in adipocytes [3], [4].

Obesity, mainly characterized by the accumulation of a large amount of fat in adipose tissues, is strongly associated with enhanced lipolytic response [5]. Moreover, increased oxidative stress has been well-documented in obesity and associated metabolic disorders and plays a central role in the pathogenesis of these disease processes [6]. Oxidative stress increases lipid peroxidation and lipid peroxides induce a variety of cellular damage directly or indirectly by covalently modifying membrane-associated or intracellular proteins. 4-HNE, derived from peroxidation of n-6 polyunsaturated fatty acids such as arachidonic and linoleic acids, is one of the most abundant and active lipid peroxides. It reacts with amino acids, such as cysteine, lysine or histidine, and forms stable adducts with proteins, thereby modulating activities and/or expression of various proteins. At high levels, 4-HNE is cytotoxic to several cell types, whereas micromolar and submicromolar concentrations of 4-HNE have been shown to induce various nontoxic, cell-specific effects. Using high-fat diet induced obesity mouse model, we previously reported that obesity was associated with increased adipose tissue 4-HNE formation [7]. In both 3T3-L1 and primary mouse adipocytes, 4-HNE treatment at nontoxic concentrations decreased adiponectin secretion via an ubiquitin-proteasome regulated mechanism [7].

This study aimed to investigate the effects of 4-HNE accumulation on lipolytic response in adipocytes. We demonstrated that 4-HNE enhanced lipolytic response in adipocytes in both basal and isoproterenol-stimulated states. Our studies revealed that both strengthened cAMP/PKA signaling and suppressed AMPK activation contributed to 4-HNE-induced lipolysis.

## Materials and Methods

### Chemicals and Antibodies

Antibiotics were purchased from Cellgro (Manassas, VA). U0126, Isoproterenol Hydrochloride, Isobutylmethylxanthine, Dexamethasone, Insulin, Oil Red O, and albumin-bound oleate were purchased from Sigma (St. Louis, MO). 4-HNE, AICAR and Glycerol assay kit were purchased from Cayman Chemical Company (Ann Arbor, MI). Free fatty acid assay kit and cAMP assay kit were purchased from BioVision (Mountain View, CA). LDH assay kit and Triglycerides assay kit were purchased from Thermo scientific (Middletown, VA). CycLex AMPK Kinase assay ELISA kit was purchased from Cyclex (Nagano, Japan). Adenylyl Cyclase Type V Inhibitor, NKY80, H89 and SB203580 were purchased from EMD Millipore (Billerica, MA USA). SP600125 and SQ22536 were purchased from Tocris (Bristol, UK). Rabbit anti-p-PDE3B (amino acid 948) polyclonal antibody was purchased from FabGennix Inc. (Frisco, TX). Rabbit anti-tubulin, actin and PDE3B polyclonal antibody were purchased from Santa Cruz Biotechnology (Dallas, TX). Other antibodies such as rabbit anti-p-AMPK (Thr172), p-HSL (ser563), p-HSL(Ser565), p-P38 (Thr180/Tyr204), p38 polyclonal antibody, rabbit anti-p-PKA (Thr197), AMPK, p-ERK1/2 (Thr202/Tyr204), ERK1/2, JNK monoclonal antibody and mouse anti-p-JNK (Thr183/Tyr185) monoclonal antibody were all purchased from Cell Signaling Technology (Danvers, MA).

### Cell Culture and Induction of Differentiation in 3T3-L1 Cells

Mouse embryo fibroblast 3T3-L1 cells were obtained from American Type Culture Collection (Manassas, VA) and grown in Dulbecco’s modified Eagle’s medium (DMEM) containing 10% fetal bovine serum (FBS) and 1% antibiotics until confluence and induced to differentiate. Briefly, 2 days postconfluence (day 0), cells were exposed to differentiation medium containing 0.5 mM isobutylmethylxanthine, 1 µM dexamethasone, 1.67 µM insulin, and 10% FBS for 3 days. Cells were then transferred to DMEM with 1.67 µM insulin and 10% FBS and re-fed every 2 days. Maturation of adipocytes was confirmed by Oil Red O staining of lipid droplets on day 7.

### Isolation and Culture of Primary Adipocytes

Male C57BL/6 mice (8–9 weeks old) were used to obtain primary adipocytes. Male C57BL/6 mice (Charles River Laboratories, Wilmington, MA; 8 weeks old) weighing 25±0.5 g were housed in Biologic Resources Laboratory in the University of Illinois at Chicago and the studies were approved by the UIC Animal Care Committee (ACC protocol # 12-032), which is certified by the American Association of Accreditation of Laboratory Animal Care. Briefly, mice were anesthetized and euthanized via cervical dislocation. Epididymal fat pads were harvested, washed in phosphate-buffered saline (PBS, pH 7.4) at room temperature, and minced thoroughly (2–3 mm) in collagenase solution (0.2 mg/mL collagenase A; 4 mL/g of adipose tissue). This mixture was incubated at 37°C with shaking at 120 rpm for 30 minutes. After digestion, the mixture was filtered through a 250 µm gauze mesh into a 50 ml conical polypropylene tube and allowed to stand for 2–3 minutes. The floating layer of adipocytes was washed 3 times and incubated at 37°C in DMEM containing 1% bovine serum albumin.

### Measurement of Lipolysis in Human Adipose Tissue

Human visceral adipose tissue explants were washed in culture plates with pre-warmed Dulbecco’s PBS containing 100 U/mL penicillin and 100 mg/mL streptomycin. After removing connective tissue and blood vessels by dissection, 20 mg of adipose tissue explants were placed into 24-well plates, cut into small pieces, and cultured in 200 µL of Dulbecco’s modified Eagle’s medium with 2 mmol/L l-glutamine, 50 U/mL penicillin, 50 mg/mL streptomycin, and 2% fatty acid-free bovine serum albumin in the presence or absence of 4-HNE for 2 hours. Glycerols released into the culture medium were determined by a Glycerol assay kit.

### Oil Red O Staining in Adipocytes

Lipid droplets in mature adipocytes were stained with Oil Red O. Cells were fixed with 10% formalin and incubated with 60% (wt/wt) filtered Oil Red O in 100% isopropanol for 30 minutes at room temperature. Cells were then washed twice with distilled water to remove excess dye and photographed under microscopy.

### Glycerol Assay

Glycerol content in the culture medium of adipocytes served as an index of lipolysis and was determined at absorption at 540 nm by use of a colorimetric assay kit.

### Free Fatty Acid Assay

The concentration of FFAs in the culture medium was determined by colorimetric assay. Briefly, 50 µL of culture medium were mixed with 2 µL of a FFAs probe and 2 µL of enzyme mixture and the reaction was developed for 30 minutes at 37°C. Absorbance at 570 nm was spectrophotometrically measured in a 96-well plate.

### Intracellular cAMP Determination

Differentiated 3T3-L1 adipocytes were exposed to 4-HNE for indicated periods. Adipocytes were then washed with PBS and lysed in 0.1 mM hydrochloric acid. After centrifuging at 600 g at room temperature, supernatants were used to measure cAMP contents with a cAMP assay kit. We used the rest of the adipocytes in each group to quantify protein content by the Bradford method for equal loading among different groups.

### Measurement of Intracellular TG Content

To determine the intracellular TG content, adipocytes seeded in 24-well plates were washed twice with phosphate buffered saline (PBS) and cellular lipids were extracted by1 ml hexane : isopropanol (3∶2) mixture. Centrifuge at 10,000 for 5 mins. Transfer upper liquid (20 microL) into new tubes. Use Speedvac to remove solvents for 20∼30 mins. Add TG Buffer Solution (Triglycerides Assay Kit, 600 microL/tube) and incubate at 37°C for 5 mins. TG content was determined by enzymatic colorimetric methods, and read absorbance at 505 nm. Cells undergoing the same treatment conditions were lysed in RIPA buffer for protein concentration determination and data normalization.

### Lactate Dehydrogenase Assay

Cell death was determined by the measurement of lactate dehydrogenase (LDH) release into the culture medium. LDH activity was determined spectrophotometrically at 340 nm using a commercially available kit (Thermo scientific, Middletown, VA).

### ELISA (Enzyme-Linked Immunosorbent Assay) Assay for AMPK Activity

After 24-hour incubation with insulin-free medium, differentiated 3T3-L1 adipocytes were exposed to 4-HNE at indicated doses for 2 hours. Adipocytes were directly lysed in extraction buffer (50 mM Tris-HCl, pH 7.5, 50 mM NaF, 5 mM sodium pyrophosphate, 1 mM EDTA, 10% glycerol, 1 mM dithiothreitol, 1% Triton X-100) and the extract centrifuged at 16, 000 × g for 10 minutes. The resulting supernatant was collected to determine intracellular AMPK activity via CycLex AMPK Kinase Assay ELISA kit according to the manufacturer’s instruction.

### Western Blotting

Fully-differentiated 3T3-L1 or primary adipocytes were lysed in RIPA buffer (containing a protease inhibitor cocktail, 2 mM Na_3_VO_4_, and 1 mM of each of the following: NaF, imidazole, Na_3_MoO_4_, Na_3_P_2_O_3_, β-glycerophosphate, and Na-tartarate) and isolated proteins were separated by SDS polyacrylamide gel electrophoresis and transferred to 0.45 microm polyvinylidene difluoride membrane. After transfer, membranes were blocked in 1% bovine serum albumin in TBS with 0.1% Tween 20 and probed with primary antibodies. Horseradish peroxidase-conjugated secondary antibodies and an enhanced chemiluminescence substrate kit were used in detection of specific proteins.

### Statistical Analysis

Data are expressed as mean ± SD. Statistical analysis was performed using a one-way ANOVA and was analyzed further by Newman-Keuls test for statistical difference. Differences between treatments were considered to be statistically significant at P<0.05.

## Results

### Increased Triglyceride Accumulation in Adipocyte is Associated with Increased Intracellular 4-HNE Contents

**Figure 1:**
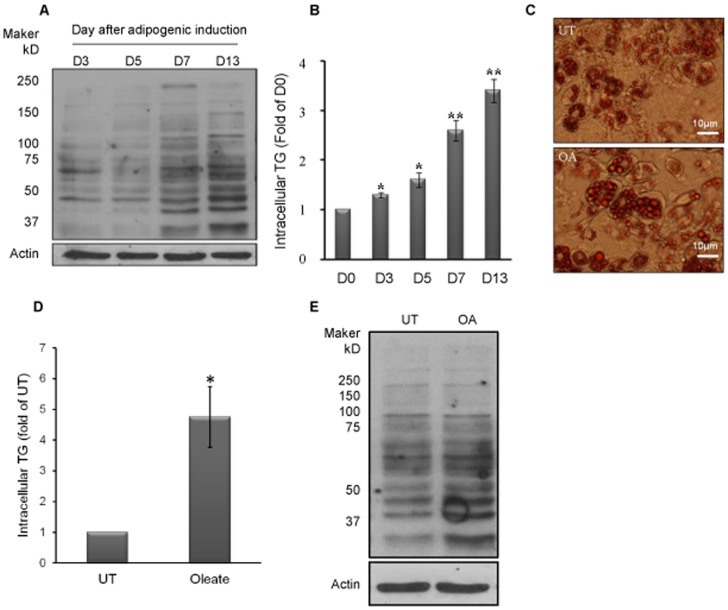
Increased triglyceride accumulation in adipocyte is associated with increased intracellular 4-HNE contents. A, Adipogenesis in 3T3-L1 fibroblasts was induced by standard adipogenesis-inducing mix. Intracellular 4-HNE-protein adducts formation were detected by Western blot on day 3, 5, 7, and 13 during the process of adipogenesis. Significant elevations in intracellular 4-HNE contents were observed on day 7 (D7) in comparison to these at day 3 and 5, and the elevations continued to day 13 (D13). B, Adipogenesis in 3T3-L1 fibroblasts was induced by standard adipogenesis-inducing mix. Time-course changes of intracellular triglyceride (TG) levels were determined on day 0, 3, 5, 7, and 13 during the process of adipogenesis. All values are denoted as Means ± SD from three or more independent batches of cells. *p<0.05 vs. D0. C & D, Oleate, at the concentration of 0.2 mM was next added into the culture medium on day 6 after the induction of adipogenesis and TG contents were determined 24 hours later (day 7) by Oil Red O Staining and biochemical assay in adipocytes as described in the materials and method. UT, untreated 3T3-L1 cells (day6); OA, Oleate. All values are denoted as Means ± SD from three or more independent batches of cells. *p<0.05 vs. D6. D, Oleate, at the concentration of 0.2 mM was next added into the culture medium on day 6 after the induction of adipogenesis and intracellular 4-HNE adducts formation was determined by Western blot on day 7. UT, untreated 3T3-L1 cells (day6); OA, Oleate.

Obesity is mainly originated from adipocyte/adipose tissue enlargement due to increased intracellular TG accumulation. We previously reported that the obesity induced by long-term high-fat diet feeding was associated with increased adipose tissue 4-HNE contents [7]. To further determine this association, we first examined time-course changes of intracellular 4-HNE contents during the process of adipogenesis using 3T3-L1 fibroblasts as an *in vitro* model. Adipogenesis was induced by standard adipogenesis-inducing mix and whole cell lysate samples were collected at day 3, 5, 7, and 13, respectively, and subjected to Western blot for the detection of intracellular 4-HNE-protein adducts formation. As shown in Fig. 1, the significant elevations in intracellular 4-HNE contents were observed at day 7 (fully differentiated) in comparison to these at day 3 and 5, and the elevations continued to day 13 (Fig. 1A). The increased intracellular 4-HNE formation was corresponded to significantly increased TG accumulation (Fig. 1B). To further verify the existence of positive correlation between intracellular TG accumulation and 4-HNE formation, oleate, a 16-C monounsaturated fatty acid, at the concentration of 0.2 mM was next added into the culture medium at day 6 and intracellular 4-HNE and TG contents were determined 24 hours later (day 7). As shown in Fig. 1C & D, the addition of exogenous fatty acid increased intracellular TG accumulation, which was accompanied by elevated intracellular 4-HNE formation (Fig. 1E), indicating that increased cellular TG accumulation is associated with elevated 4-HNE formation in adipocytes.

### 4-HNE Activates Lipolysis in Adipocytes

**Figure 2:**
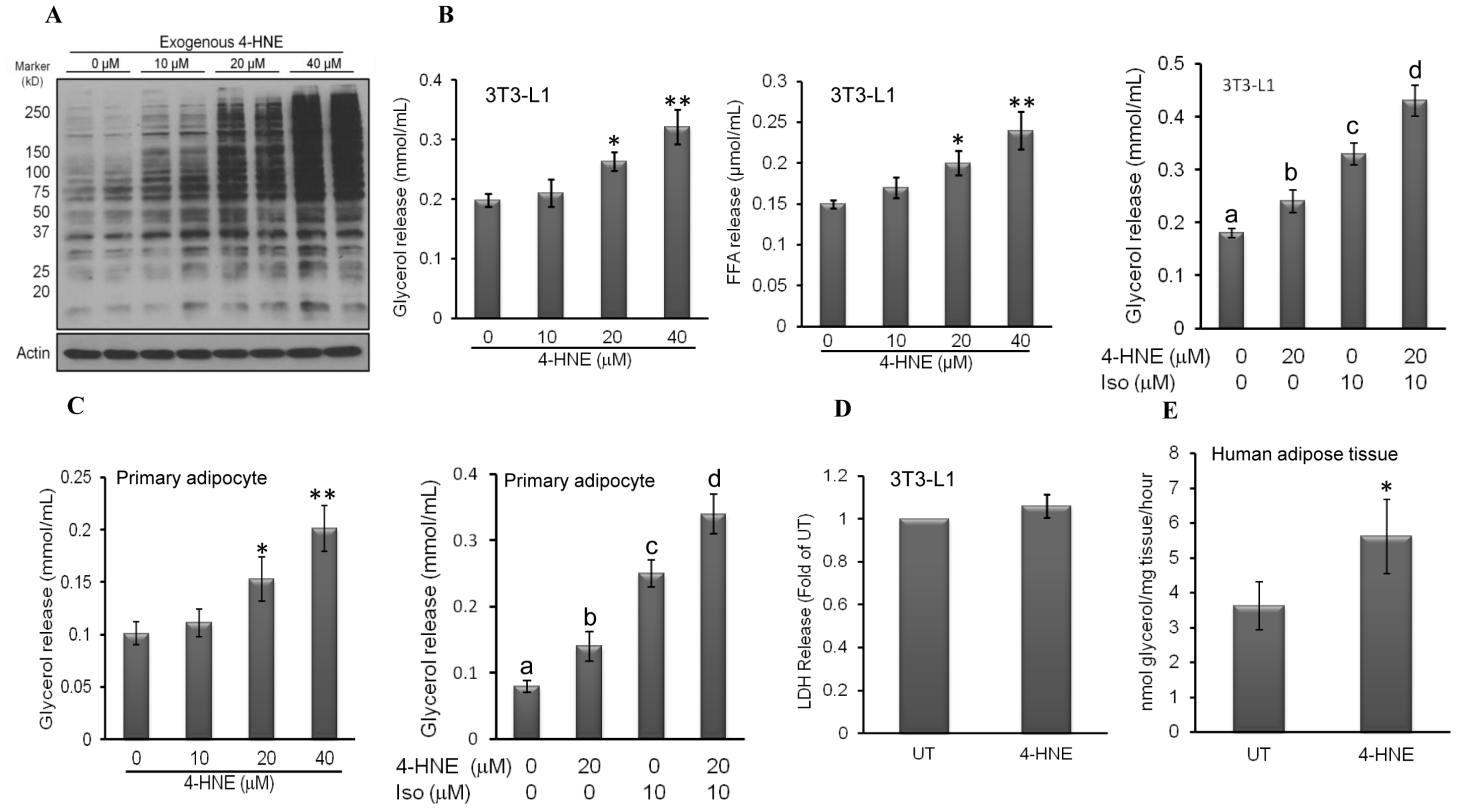
4-HNE activates lipolysis in adipocytes. A, Fully-differentiated 3T3-L1 adipocytes were treated with 4-HNE (0, 10, 20, and 40 microM) for 4 hours, intracellular 4-HNE contents were determined by Western blot. B, Fully-differentiated 3T3-L1 adipocytes were stimulated with isoproterenol in the presence or absence of 4-HNE pretreatment for 5 hours. Glycerol and fatty acids releases were measured. All values are denoted as Means ± SD from three or more independent batches of cells. *p<0.05 and **p<0.01 vs. untreated cells. Bars with different characters differ significantly (p<0.05). C, Primary mouse adipocytes were freshly isolated from epididymal fat pad and stimulated with isoproterenol in the presence or absence of 4-HNE pretreatment for 5 hours. Glycerol releases were measured. All values are denoted as Means ± SD from three or more independent batches of cells. *p<0.05 and **p<0.01 vs. untreated cells. Bars with different characters differ significantly (p<0.05). D, Fully-differentiated 3T3-L1 adipocytes were treated with 40 microM 4-HNE for 5 hours. LDH activities in the media were measured. All values are denoted as Means ± SD from three or more independent batches of cells. *p<0.05 vs. untreated cells. E, Minced human visceral adipose tissue was treated with/without 40 microM 4-HNE for 4 hours and glycerol levels in the media were measured. All results are mean ± SD from at least three independent experiments (n ≥3), *p<0.05 vs. untreated adipose tissues.

Enhanced lipolytic response and subsequent increase of circulating FFAs is one of the major features in obesity-related disorders. To investigate whether increased intracellular 4-HNE in adipocyte causes enhanced lipolysis, we measured the glycerol and fatty acids releases from fully-differentiated 3T3-L1 and primary mouse adipocytes exposed to exogenous 4-HNE. In consistent with our previous report [7], exogenous 4-HNE exposure increased intracellular 4-HNE accumulation in a dose-dependent fashion (Fig. 2A). In fully-differentiated 3T3-L1 adipocytes, a 5-hour 4-HNE exposure elevated lipolytic reaction in a dose-dependent manner at both basal and isoproterenol-stimulated conditions, evidenced by significantly increased glycerol and fatty acids releases in comparison to control adipocytes (Fig. 2B). The similar results were observed when primary mouse adipocytes were used (Fig. 2C). At this time point, 4-HNE at highest concentration used in this study (40 microM) did not affect adipocyte viability, assayed by LDH release (Fig. 2D). To evaluate *ex vivo* lipolytic action, minced human visceral adipose tissue was treated with/without 40 microM 4-HNE for 4 hours and glycerol levels in the media were measured. As shown in Fig. 2E, glycerol release was significantly increased in 4-HNE exposed adipose tissues.

### 4-HNE Enhances Lipolytic Response in Adipocyte via Activating the cAMP/PKA Signaling

**Figure 3:**
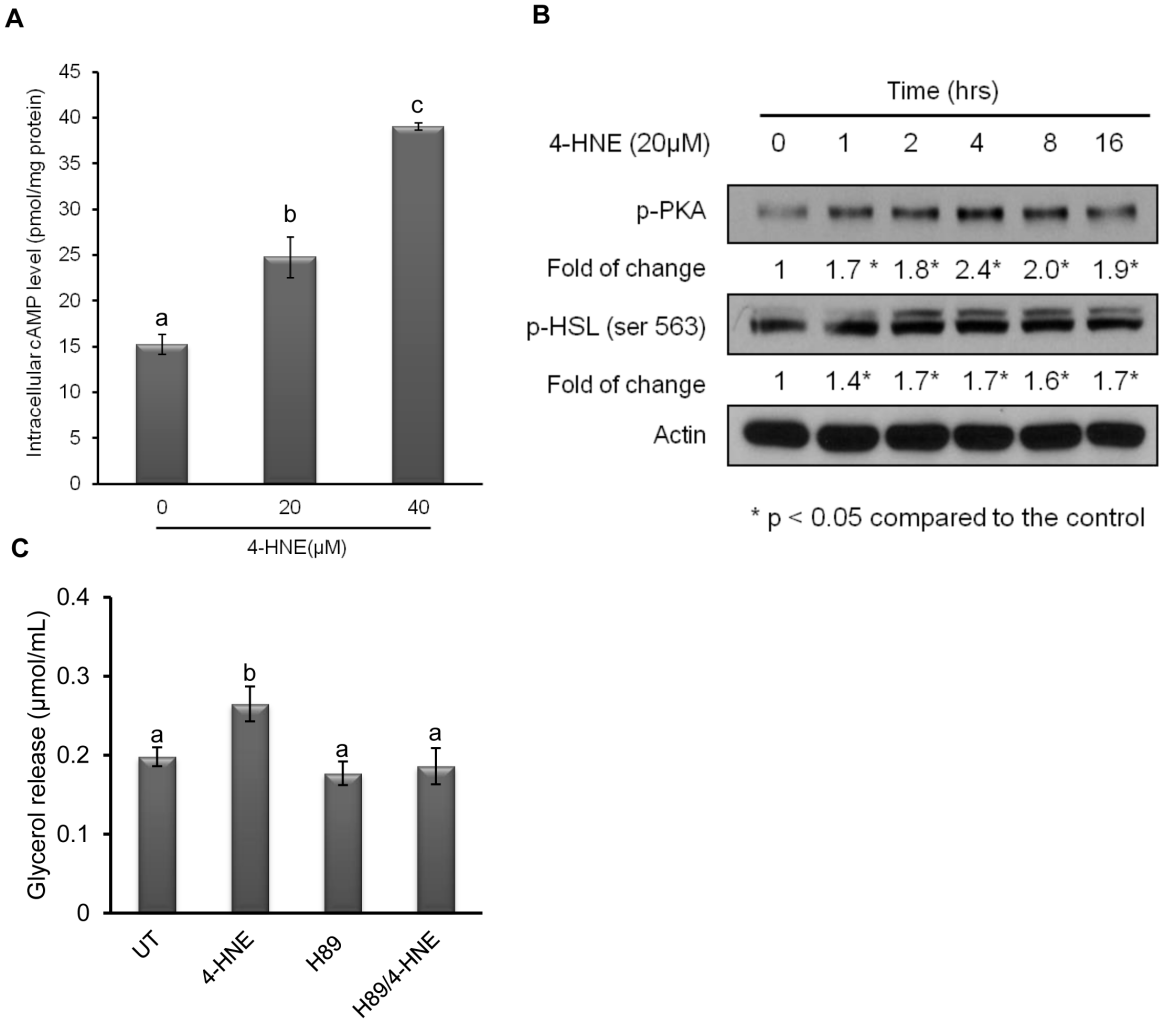
4-HNE induces lipolysis via activating the cAMP/PKA signaling. A, Fully-differentiated 3T3-L1 cells were incubated with 4-HNE (20 and 40 microM) for 1 hour. Intracellular cAMP levels were measured by ELISA assay. All values are denoted as Means ± SD from three or more independent batches of cells. Bars with different characters differ significantly (p<0.05). B, Fully-differentiated 3T3-L1 cells were incubated with 4-HNE at 20 µM for indicated time points. Western blot was conducted to determine phosphorylated PKA and HSL protein abundance. The degree of changes (fold) was calculated as the optimal density ratio of the phosphorylated target protein to the corresponding total protein, normalized to the control cells. C, Fully-differentiated 3T3-L1 cells were pre-incubated with H89 (5 microM) for 2 hours before 4-HNE exposure (20 microM). Glycerol releases were measured 16 hours later. All values are denoted as Means ± SD from three or more independent batches of cells. Bars with different characters differ significantly (p<0.05).

**Figure 4:**
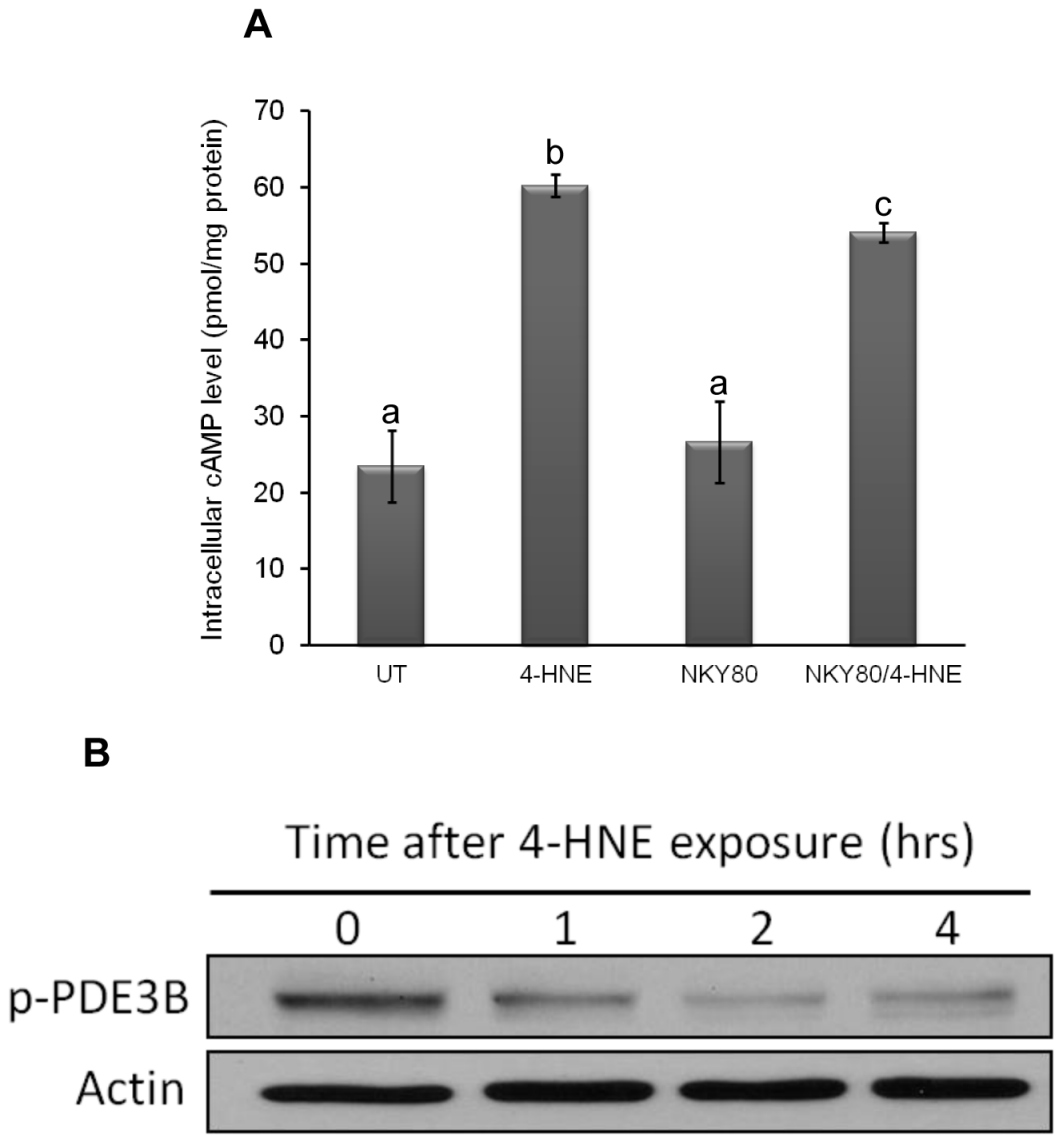
Both adenylyl cyclase activation and PDE3B suppression contribute to 4-HNE- induced intracellular cAMP elevation in adipocytes. A, Fully-differentiated 3T3-L1 cells were pretreated with NKY80 (20 microM) for 1 hour, followed by 4-HNE exposure for 1 hour. Glycerol releases were determined. All values are denoted as Means ± SD from three or more independent batches of cells. Bars with different characters differ significantly (p<0.05). B, Fully-differentiated 3T3-L1 cells were exposed to 4-HNE (20 microM) for indicated time points. Western blot was performed to detect phosphorylated PDE3B protein abundance.

The cAMP/PKA system is the major signaling pathway involved in the regulation of lipolysis. Lipolytic inducers, such as glucagon and catecholamines, increase intracellular cAMP levels via binding to G protein-coupled receptors. As a consequence, cAMP activates PKA, which phosphorylates (and activates) HSL. To determine the implication of this pathway in the 4-HNE-induced lipolytic reaction, we first measured intracellular cAMP levels by ELISA assay. As shown in Fig. 3A, 4-HNE exposure quickly increased intracellular cAMP levels (1 hour) in a dose-dependent manner. Since PKA is the direct downstream kinase activated by increased intracellular cAMP levels, we next examined PKA activation by Western blot using antibody detecting phosphorylated PKA. As shown in Fig. 3B & C, incubation with 20 microM 4-HNE quickly induced PKA phosphorylation, peaking at 4 hour after 4-HNE exposure. Accompanying increased PKA phosphorylation, HSL (ser563) phosphorylation was upregulated. Pre-incubation with H89 (dissolved in DMSO, 5 microM), a potent PKA inhibitor, markedly attenuated 4-HNE stimulated glycerol release (Fig. 3C), suggesting that lipolytic response to 4-HNE is mediated mainly by cAMP/PKA signal pathway in adipocytes. Intracellular cAMP levels are controlled by both adenylyl cyclase (for synthesis) and phosphodiesterase (PDE), with phosphodiesterase type-3B (PDE3B) being the major isoform in adipocytes (degradation). Insulin exerts its anti-lipolytic effect mainly via phosphorylating/activating PDE3B activity, thereby decreasing intracellular cAMP levels [8]. Inhibition of adenylyl cyclase with its specific inhibitor, NKY80, partially prevented 4-HNE-induced increase of intracellular cAMP (Fig. 4A). Furthermore, 4-HNE decreased phosphorylated PDE3B protein abundance in response to 4-HNE exposure (Fig. 4B), suggesting that both adenylyl cyclase activation and PDE3B suppression contributes to 4-HNE induced intracellular cAMP elevation in adipocytes.

### MAP Kinases Activation is not Involved in 4-HNE Elicited Lipolytic Response

**Figure 5:**
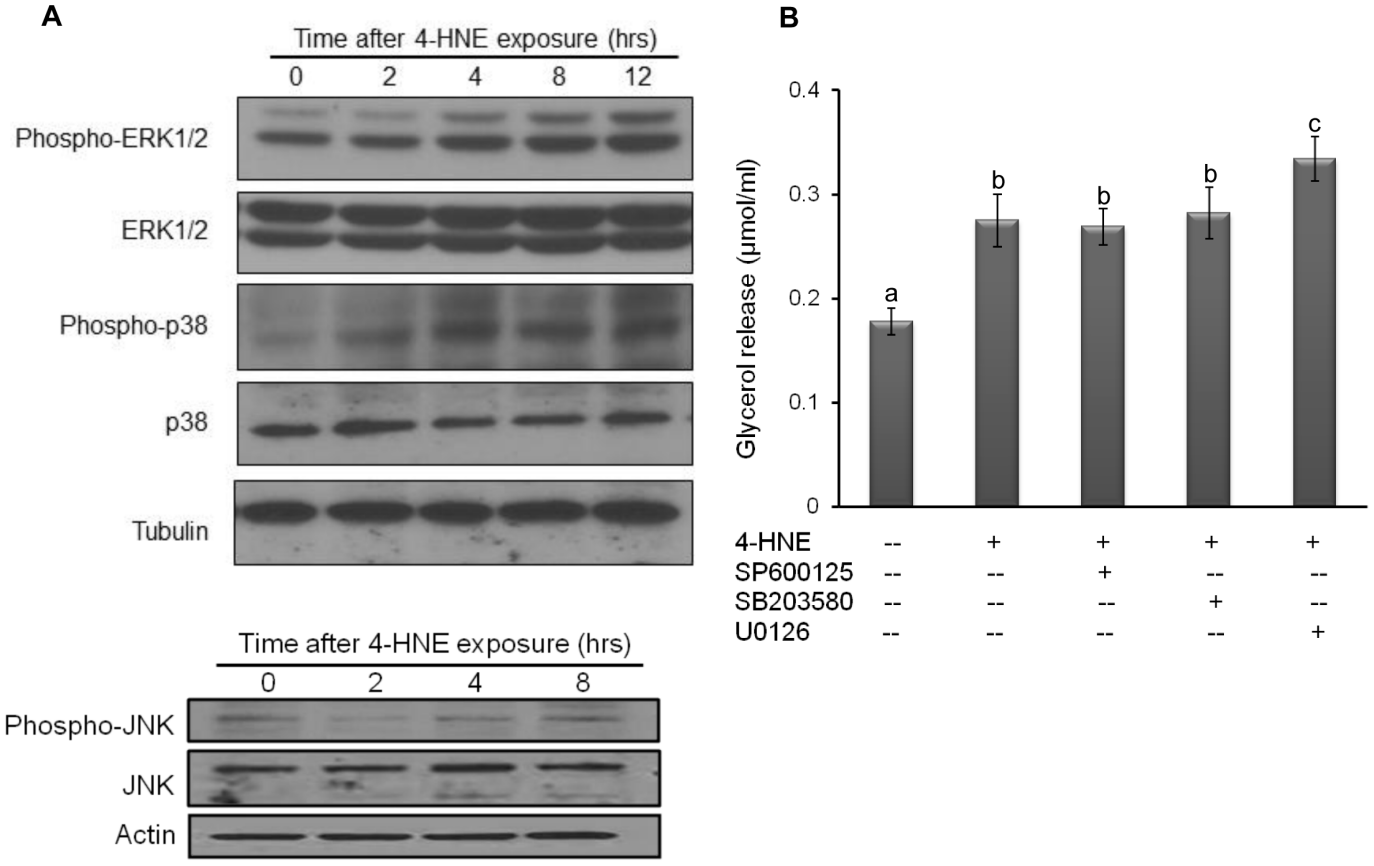
MAP kinases activation is not involved in 4-HNE-elicited lipolytic response. A, Fully-differentiated 3T3-L1 cells were treated with exogenous 4-HNE (20 microM) for indicated time points. The activations of the MAP kinases, including ERK1/2, p38, and JNK, were detected by immunoblotting. B, Fully-differentiated 3T3-L1 cells were pretreated with specific kinase inhibitors for ERK1/2 (U0126, 10 microM), JNK (SP600125, 10 microM), and p38 (SB203580, 10 microM) for 1 hours, followed by exogenous 4-HNE (20 microM) exposure. Glycerol releases were measured 6 hours later. All values are denoted as Means ± SD from three or more independent batches of cells. Bars with different characters differ significantly (p<0.05).

In addition to the cAMP/PKA pathway, the activation of the MAP kinases, including ERK1/2, p38, and JNK, are also reported to promote lipolysis. To determine whether MAP kinases are involved in 4-HNE induced lipolytic response, we first examined the effect of 4-HNE exposure on phosphorylation/activation of three MAP kinases, respectively. Immunoblotting analysis showed that the exposure to 20 microM 4-HNE quickly activated ERK1/2 and p38 pathways, whereas JNK activation was not affected (Fig. 5A). Based on these observations, we next examined the effects of specific kinase inhibitor of three MAP kinases on 4-HNE-induced lipolytic response via pre-incubating (1 hour) fully-differentiated 3T3-L1 adipocytes with U0126 (10 microM, for MEK/ERK1/2), SP600125 (10 microM, for JNK), and SB203580 (10 microM, for p38), respectively, prior to 4-HNE exposure for another 6 hours. As shown in Fig. 5B, neither p38 nor JNK inhibitors impacted 4-HNE-induced lipolytic enhancement. Interestingly, ERK1/2 inhibition aggravated 4-HNE induced increase of glycerol release, suggesting that 4-HNE induced lipolytic response is independent of MAP kinases activation.

### Inhibition of AMPK Contributes 4-HNE Induced Lipolytic Response

**Figure 6:**
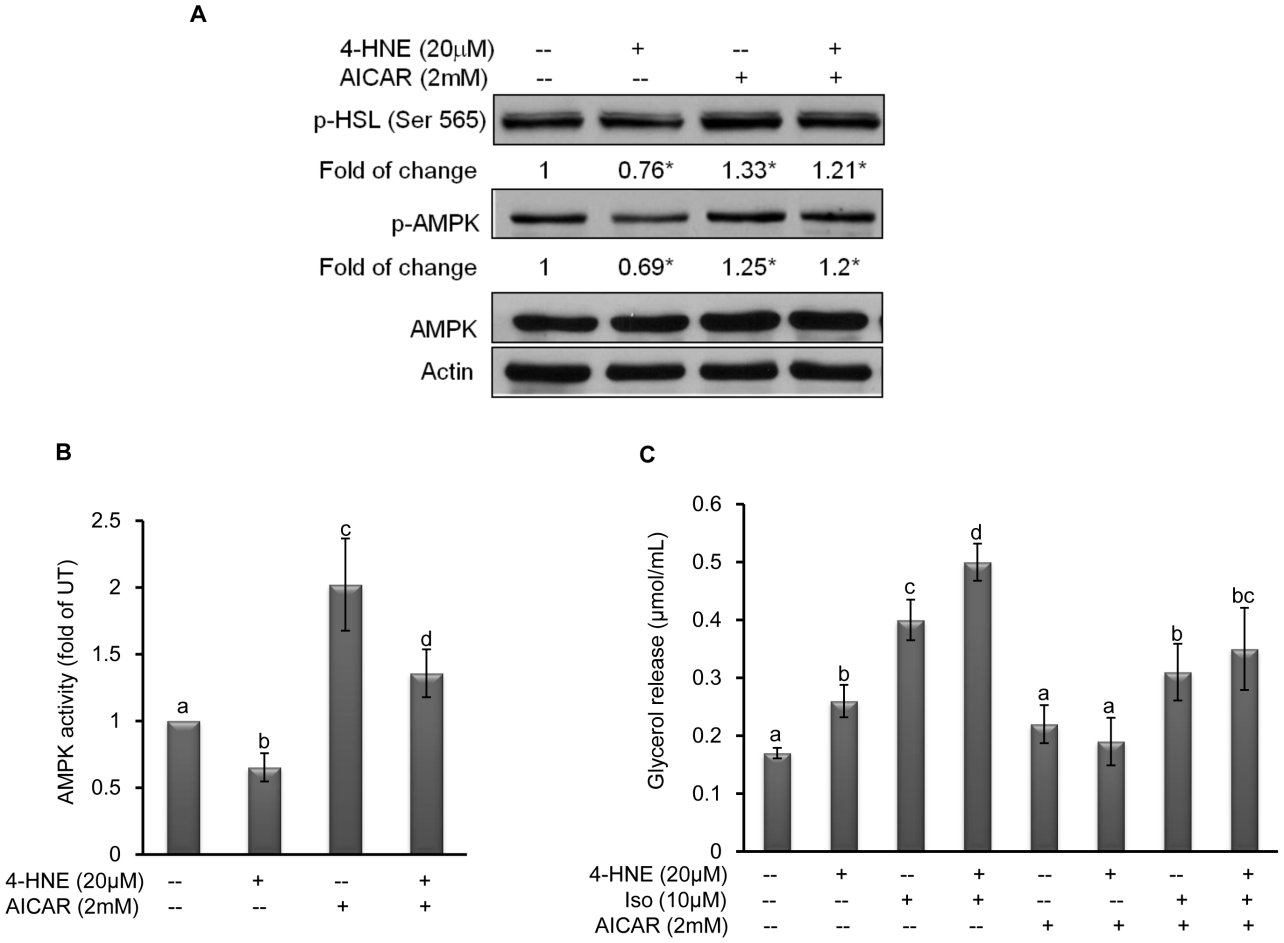
Inhibition of AMPK contributes 4-HNE-induced lipolytic response. A, Fully-differentiated 3T3-L1 cells were treated with exogenous 4-HNE (20 microM) in the presence or absence of AICAR inclusion in the media for 2 hours. AMPK phosphorylation and HSL phosphorylation at serine 565 were examined. The degree of changes (fold) was calculated as the optimal density ratio of the phosphorylated target protein to either actin or the corresponding total protein, normalized to the control cells. B, Fully-differentiated 3T3-L1 cells were treated with exogenous 4-HNE (20 microM) in the presence or absence of AICAR inclusion in the media for 2 hours. AMPK activities were determined by ELISA kit. All values are denoted as Means ± SD from three or more independent batches of cells. Bars with different characters differ significantly (p<0.05). C, Fully-differentiated 3T3-L1 adipocytes were first treated with AICAR, an AMPK inducer, for 2 hours before 4-HNE exposure. Glycerol release with/without isoproterenol stimulation was determined 5 hours later. All values are denoted as Means ± SD from three or more independent batches of cells. Bars with different characters differ significantly (p<0.05).

In contrast to conventional cAMP/PKA and MAP kinases pathways, previous studies established that AMPK activation suppressed lipolytic response at both basal and isoproterenol-stimulated states via phosphorylating HSL at ser565 site, thereby inhibiting its activity [9]. Inhibitory effect of 4-HNE on AMPK was reported in cardiac myocytes previously [10]. To determine whether AMPK suppression also contributes to 4-HNE-induced enhancement in lipolysis in adipocytes, we first examined the effects of 4-HNE (20 microM) on phosphorylation status of AMPK and HSL at ser565 by Western blot. As shown in Fig. 6A & B, a 2-hour exposure to 4-HNE suppressed AMPK phosphorylation and enzymatic activity. Inclusion of AICAR (2 mM), an AMPK activator, in the media activated AMPK and prevented 4-HNE-induced AMPK suppression. In response to suppressed AMPK activation, HSL phosphorylation at ser565 was also suppressed. In contrast, AICAR alone enhanced HSL (ser565) phosphorylation and prevented 4-HNE-induced suppression of HSL (ser565) phosphorylation. To further determine the role of AMPK suppression in 4-HNE-induced lipolysis, fully-differentiated 3T3-L1 adipocytes were treated with AICAR for 2 hours before 4-HNE exposure. Glycerol release with/without isoproterenol (10 µM) stimulation was determined 5 hours later. As shown in Fig. 6C, AICAR decreased glycerol release in comparison to untreated adipocyte and attenuated 4-HNE induced lipolysis at both basal and isoproterenol-stimulated states.

## Discussion

Adipose tissue oxidative stress plays a central/causal role of in the pathogenesis of metabolic syndrome [11]–[14]. Intracellular fat contents in adipocytes are positively associated with lipid peroxidation. 4-HNE is one of the most abundant and reactive aldehydic products derived from peroxidation of n-6 polyunsaturated fatty acids [15]–[17]. We previously reported that the obesity induced by high-fat diet feeding in mice was associated with elevated adipose tissue 4-HNE contents [7]. In the present study, we further demonstrated that intracellular TG accumulation in adipocytes is positively correlated with intracellular 4-HNE production. Notably, the exposure of adipocytes to exogenous 4-HNE, which increased intracellular 4-HNE content, leads to enhanced lipolytic response under both basal and isoproterenol-stimulated conditions. Mechanistic investigations reveal that both cAMP/PKA activation and AMPK suppression contribute to 4-HNE-induced enhancing effect on lipolysis. Although the enhanced lipolytic activation can serve as an adaptive response that regulates energy homeostasis when facing energy overconsumption, chronic oxidative stress and sustained 4-HNE elevation could contribute to lipotoxicity and impair insulin sensitivity in other tissues, such as liver and muscle, because of persistently accelerated FFA efflux from adipocytes to the bloodstream.

Unlike originally thought, adipocytes are in fact highly reactive to oxidative stress. Even under resting conditions, lipid peroxidates, such as 4-HNE, can be detected in adipocytes [18], [19]. Oxidative stress induced by glucose oxidase exposure in 3T3-L1 adipocytes doubled the intracellular 4-HNE levels [18], [19]. Furthermore, high-fat diet feeding was associated with increased adipose tissue protein carbonylation [20]. In the present study, we observed a time-course increases of intracellular 4-HNE contents during adipogenesis, which was correlated with intracellular TG accumulation. Moreover, increased intracellular TG accumulation by the inclusion of oleic acid in the media resulted in 4-HNE accumulation in fully-differentiated 3T3-L1 adipocytes, suggesting that increased TG content plays a causal role in the induction of oxidative stress and lipid peroxidation in adipocytes. At this point, it is difficult to distinguish the individual contribution of TG accumulation and oxidative stress to the observed lipolytic response. N-acetylcysteine (NAC) supplementation to fully-differentiated 3T3-L1 adipocytes suppressed lipolysis induced by oleic acid, however, it also reduced intracellular TG accumulation (unpublished data).

Our findings in this study suggested that the mechanisms by which 4-HNE stimulates lipolysis are multifactorial. Increase in intracellular cAMP contents and subsequent cAMP/PKA pathway activation is the major early signals that control lipolysis with catecholamine stimulation [21], [22]. Here, our results documented for the first time that 4-HNE exposure quickly elevated intracellular cAMP contents in adipocytes. The subsequent increase in PKA phosphorylation/activation and the observation that H89, a specific PKA inhibitor, inhibited the lipolytic response induced by 4-HNE exposure, suggest that the activation of this conventional lipolytic pathway plays an important role in this process. In general, intracellular cAMP level is controlled by both adenylyl cyclase and phosphodiesterase (PDE), with PDE3B being the major isoform in adipocytes [23]–[25]. cAMP is synthesized from ATP by adenylyl cyclase located on the inner side of the plasma membrane and metabolized into AMP by phosphodiesterase [26]. Adenylyl cyclase is activated by a range of signaling molecules through the activation of adenylyl cyclase stimulatory G (G_s_)-protein-coupled receptors and inhibited by agonists of adenylyl cyclase inhibitory G (G_i_)-protein-coupled receptors [26]. Insulin exerts its anti-lipolytic action via the phosphorylation and subsequent activation of PDE3B [27]. The activation of this enzyme is accompanied by a decrease in intracellular cAMP, whereas its suppression is always associated with increased intracellular cAMP levels [28]. In the present study, adenylyl cyclase inhibitors attenuated 4-HNE-induced lipolysis. Additionally, PDE3B phosphorylation was suppressed in response to 4-HNE exposure, implying that both adenylyl cyclase activation and PDE3B suppression contribute to 4-HNE induced intracellular cAMP elevation. Although the current study did not provide clear mechanistic explanation in terms of how 4-HNE affects these enzymes, given the fact that 4-HNE can actively modify proteins by forming covalent adduct, it is rational to postulate that 4-HNE may form protein adduct with these enzymes, thereby altering their functions. The future investigation is certainly warranted.

Mitogen-activated protein kinases (MAP kinases) are serine/threonine-specific protein kinase, consisting of ERK1/2, p38, and JNK. The participation of the individual MAP kinase in lipolytic process has been reported [29]. The effects of 4-HNE on MAP kinases are kinase-, stimulus-, and cell type-dependent. In adipocytes, 4-HNE activated p38, leading to increased COX-2 expression and inflammation [30]. In hepatocytes, 4-HNE suppressed ERK1/2 activation [31], whereas it activated ERK1/2 in endothelial cells [32]. Furthermore, 4-HNE-induced JNK activation contributed to cellular dysfunction and apoptosis in a variety of cell types [33]. In consistent with previous studies, our data showed that 4-HNE activated ERK1/2 and p38 in fully-differentiated 3T3-L1 adipocytes. However, inhibition of their activities had no effects on 4-HNE-induced glycerol release, excluding the potential involvement of MAP kinases activation in 4-HNE-induced lipolytic activation.

AMPK is a ubiquitously expressed serine/threonine kinase and functions as an intracellular energy sensor. It is activated in response to elevated intracellular AMP/ATP ratio [3], [34], leading to increased glucose uptake and fatty acid oxidation [35], [36]. Accumulated evidence supports that AMPK activation in adipocytes confers inhibitory effect on lipolysis in adipocytes [4], [37], [38]. Activation of AMPK either chemically or genetically led to inhibited isoproterenol-stimulated lipolytic processes [38], [39]. Conversely, AMPK inhibition enhanced lipolytic response in adipocytes [40], [41]. The activation of AMPK by isoproterenol, due to increased fatty acid re-absorption and metabolism, is considered to be a feedback-inhibitory mechanism in regulating the lipolytic process [42]. In retinal pigment epithelium (RPE) cells, 4-HNE caused cell death via down-regulating basal activity of AMPK, which involved decreased Thr172 phosphorylation of AMPKα [43]. In the present study, we observed that 4-HNE exposure was associated with suppressed AMPK activation in adipocytes. The attenuation of 4-HNE-induced lipolysis by AICAR, an AMPK activator, suggests that AMPK suppression participated in this process. AMPK activation was reported to increase phosphorylation of Ser565 on HSL, thereby reducing the translocation of endogenous HSL to the lipid droplet to initiate the lipolytic process [37], [44]. To gain insight into the potential involvement of AMPK system, HSL phosphorylation at Ser565 was examined after 4-HNE exposure and our results confirmed that 4-HNE exposure decreased HSL phosphorylation at this site, indicating that the AMPK system is indeed a contributor in the lipolytic action induced by 4-HNE.

**Figure 7:**
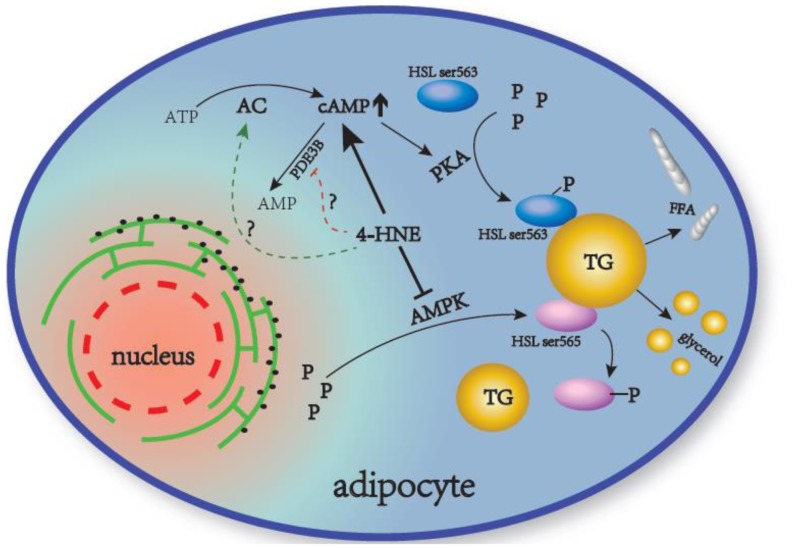
Schematic representation of the proposed mechanism underlying 4-HNE-induced enhancement of lipolytic response in adipocytes. 4-HNE-triggered lipolysis in adipocytes involves the activation of the cAMP/PKA/HSL pathway as well as the suppression of the AMPK pathway. Both AC activation and PDE3B inhibition potentially contribute to 4-HNE-induced increase of intracellular cAMP levels. PKA, protein kinase A; HSL, hormone-sensitive lipase; AMPK, AMP-activated protein kinase; P, phosphorylation; TG, triglyceride; FFA, free fatty acids; AC, adenylyl cyclase; PDE3B, phosphodiesterase 3B; ? hypothetic mechanism.

In conclusion, our findings reveal that obesity-related 4-HNE accumulation in adipocytes enhances lipolytic response in adipocytes via activating cAMP/PKA/HSL pathway and suppressing AMPK activation (Fig. 7). The enhanced lipolysis response to 4-HNE promotes FFA efflux from adipocytes to the plasma, which could be a cellular basis of obesity-related disorders, including lipotoxicity, dyslipidemia, insulin resistance, and diabetes. Moreover, because TG accumulation can trigger lipid peroxidation in many other tissues/organs, such as the liver and pancreatic beta-cells [45]–[47], the increased FFA release due to enhanced lipolysis in 4-HNE-accumulated adipocytes may produce a feed-forward mechanism to further stimulate or worsen lipid peroxidation in other tissues. This phenomenon implicates a central role of adipose tissue oxidative stress in the development of obesity-related pathologies in various tissues and organs.
